# How and why do French medical students choose the specialty of infectious and tropical diseases? A national cross-sectional study

**DOI:** 10.1186/s12909-020-02317-9

**Published:** 2020-10-31

**Authors:** Nathan Peiffer-Smadja, François-Daniel Ardellier, Pauline Thill, Anne-Lise Beaumont, Gaud Catho, Lindsay Osei, Vincent Dubée, Alexandre Bleibtreu, Adrien Lemaignen, Michaël Thy

**Affiliations:** 1 RéJIF, Young French Infectious Diseases Physicians’ Network - Réseau des Jeunes Infectiologues Français, Paris, France; 2 Infectious Diseases Department, Bichat-Claude Bernard Hospital, Assistance-Publique Hôpitaux de Paris, Paris, France; 3 Université de Paris, INSERM, IAME, F-75006 Paris, France; 4 Service de Radiologie II, Hôpitaux Universitaire de Strasbourg, Strasbourg, France; 5 Service de Maladies Infectieuses et Tropicales, Hôpital Bichat-Claude Bernard, Assistance-Publique Hôpitaux de Paris, Paris, France

**Keywords:** Career choice, Infectious diseases education, Perception of infectious diseases, Residency

## Abstract

**Background:**

Infectious and tropical diseases (ID) physicians are needed now more than ever to tackle existing and emerging global threats. However, in many countries, ID is not recognized as a qualifying specialty. The creation of ID residency in 2017 in France offers the opportunity to know how and why the specialty is chosen by medical students.

**Methods:**

We first analyzed the choice of specialty of all French medical students in 2017 and 2018 according to their rank at the national exam that ends medical studies. A web questionnaire was then sent in January 2019 to all ID residents in France (*n* = 100) to assess the factors influencing their choice of specialty and their career plan.

**Results:**

We analyzed the choice of 17,087 medical students. ID was the first-chosen specialty with a median national rank of 526/8539, followed by plastic surgery and ophthalmology. The questionnaire was completed by 90% of the French ID residents (*n* = 100). The most encouraging factors to choose ID were the multi-system approach of the specialty, the importance of diagnostic medicine and having done an internship in ID during medical school. The potential deterrents were the work-life balance, the workload and the salary.

**Conclusions:**

The recent recognition of ID as a qualifying specialty in France can be considered a success insofar as the specialty is the most popular among all medical and surgical specialties. Individuals who choose ID are attracted by the intellectual stimulation of the specialty but express concerns about the working conditions and salaries.

**Supplementary Information:**

The online version contains supplementary material available at 10.1186/s12909-020-02317-9.

## Background

In 2019, the World Health Organization published a list of 10 threats to global health among which 6 are related to infectious and tropical diseases (ID): the threat of a global influenza pandemic, antimicrobial resistance, Ebola and other high-threat pathogens, vaccine hesitancy, dengue, and HIV [1]. ID specialists’ consultations have been shown to decrease mortality rates and healthcare costs and antimicrobial stewardship programs have shown to reduce the number of infections with resistant bacteria [2–5]. In the context of the COVID-19, ID physicians are needed now more than ever to tackle this pandemic as well as the future ones and improve outcomes for patients [6].

However, in many countries, ID fellowship programs remain unfilled every year. In 2019, 75 out of 401 positions in ID (19%) were unfilled in the USA and only 3 medical specialties among the 68 specialties evaluated had a higher number of unfilled positions (geriatric medicine, nephrology and ultrasound medicine) [7]. Canada has also described difficulties in finding ID residents and fellows [8, 9]. Moreover, ID is still not widely recognized as a qualifying specialty in many countries including Spain or India [10, 11]. Studies in the USA have identified dissuading factors from choosing the ID specialty and potential targets for action, such as a low salary or an unsatisfying work-life balance. However, we do not know if these factors are similar across countries [12, 13].

In 2017, France undertook a reform of residency that created new qualifying specialties such as infectious and tropical diseases. From 2017 onward, a fixed number of positions in ID are opened every year in each city of France. At the end of medical school, French students take a national ranking exam and the medical and surgical specialties are chosen according to their rank: from the first ranked to the last, each medical student chooses a city and a specialty. Before this reform, ID was a subspecialty that could only be chosen after completing another residency (usually internal medicine or family medicine).

The creation of the ID residency represents an interesting opportunity to know how and why the specialty is chosen by French medical students and to explore their expectations, motivations and reservations when choosing ID.

## Methods

### Choice of ID specialty

The national ranking, the choice of city and the choice of specialty of each medical student are published every year in the French ministry of Health official journal [14]. We used the publication of the medical students’ choice of specialty in 2017 and 2018 to analyze the median rank in each specialty. We included every medical student entering residency in 2017 and 2018 in France.

### Questionnaire development and population

A web questionnaire to explore the factors influencing the choice of ID residency was developed using SurveyMonkey® by a working group of 15 ID specialists. The working group included one professor, two hospital practitioners, six fellows and six residents. The questionnaire took into account previous questionnaires on the topic [12, 13] and was adapted for the French curriculum. The questionnaire was reviewed and tested by two first-year ID residents to check for simplicity and comprehensiveness and was modified according to comments. The survey was anonymous and received approval from the French data protection authority (CNIL, registration number: 2213137).

The questionnaire was split into three parts (supplementary material). The first part collected information about demographics, medical school curriculum, expectations from ID residency and career plan. The second part focused on the motivations and reservations of ID residents when choosing the specialty. To explore this, we chose 36 factors relative to ID (Fig. 2) and asked the participants to use a slider to decide if it discouraged them or encouraged them to choose ID. The following legend was shown at the top of the slider for each factor: “strongly discouraged me to choose ID” at the far left, “did not influence my choice” in the middle and “strongly encouraged me to choose ID” at the far right. The slider position was then linked to a number between 0 (“strongly discouraged me to choose ID”) and 10 (“strongly encouraged me to choose ID”) to do the statistics and box plots. To capture other factors that could have been missed, we asked the participants if they thought of other factors that influenced their choice. The third part collected information about likely measures to increase the attractiveness of ID. We asked the participants to decide if a selection of 12 measures were very unlikely, unlikely, neutral, likely or very likely to increase the attractiveness of the specialty. We selected the 12 measures based on the literature [12, 13] and on the modifiable factors that were evaluated in the second part of the questionnaire. The 36 factors and 12 measures were displayed in a random order for each participant to avoid biases due to changes in the participant’s way of rating during the questionnaire (e.g. modification of the concentration level).

The questionnaire was sent on 29th January 2019 by email to all the residents who had chosen ID since the creation of the specialty in 2017 (*n* = 100) via the Young French Infectious Diseases Physicians’ Network (RéJIF), a working group of the French Society for Infectious Diseases (SPILF). A reminder was sent on 14th February 2019. No incentive was provided.

### Statistical analysis

Data from the survey were imported into R software (version 3.2.4). Numerical data are presented as absolute numbers, proportion, median ± interquartile range (IQR).

To represent the choice of medical and surgical specialties by the medical students, we drew a ridgeline plot which represents a superposition of density curves by choice of specialty according to the ranking of medical students in 2017 and 2018. We used the R packages “ggridges” and “ggplot2” for the ridgeline plot.

## Results

### Choice of ID specialty

**Fig. 1:**
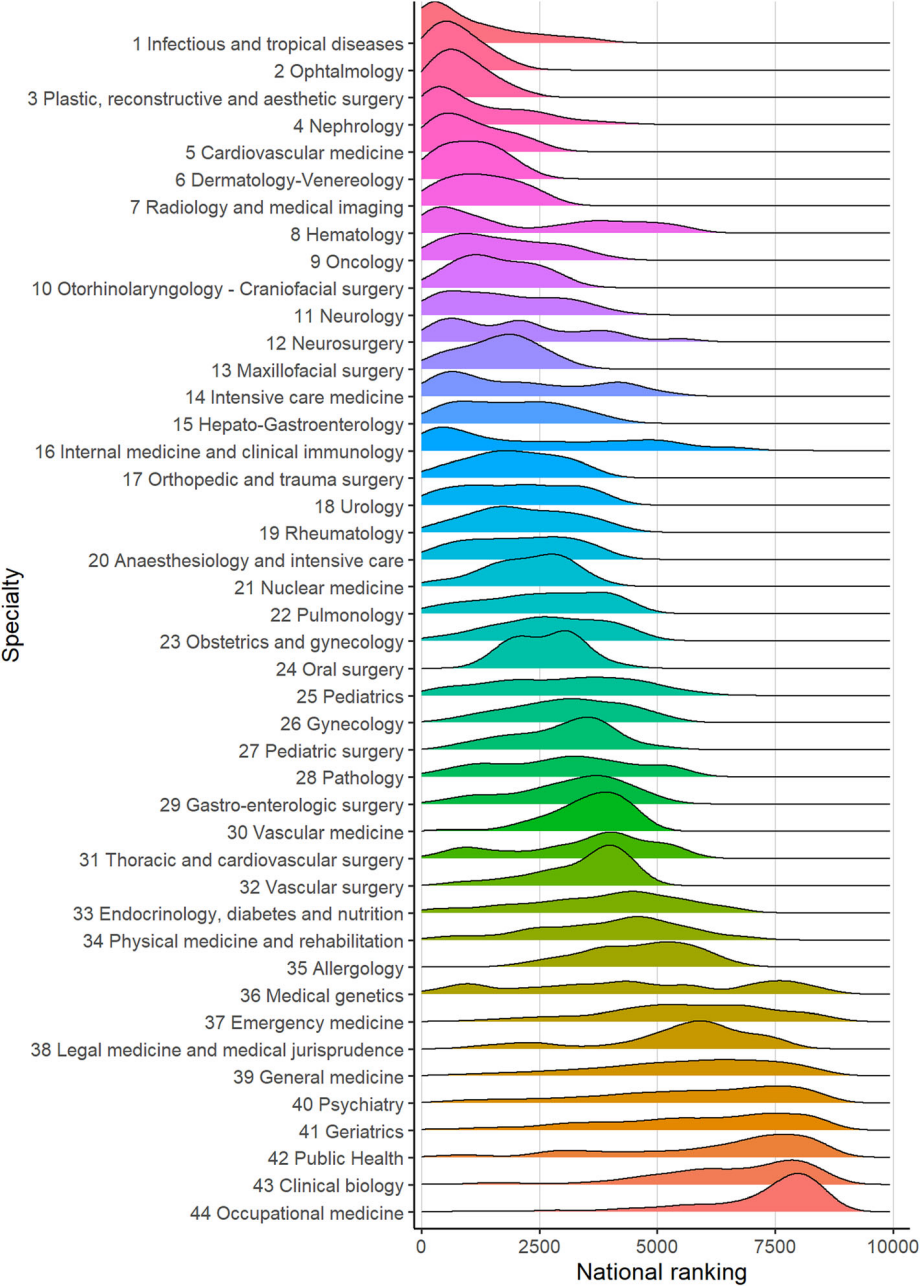
Ranking of specialties according to student ranking. Legend: Ridgeline plot of density curves by choice of specialty according to the ranking of medical students using data from 2017 and 2018. Specialties are ranked by median ranking of students at the national ranking exam

We analyzed the choice of medical or surgical specialty of 17,078 medical students: 8372 who took the French national ranking exam in 2017 and 8706 in 2018 (Table 1). Forty-nine and 51 positions for the ID specialty were respectively opened in 2017 and 2018 and all were filled. ID was the first-chosen specialty among all specialties with a mean median rank of 526/8539 (Fig. 1). Both in 2017 and in 2018, the first nationally ranked medical student chose the ID specialty. ID was not available for medical students ranked over 3709th out of 8372 in 2017 and over 3209th out of 8706 in 2018. ID specialty was followed by ophthalmology (mean median rank 604) and plastic surgery (mean median rank 723).

**Table 1:** Choice of medical and surgical specialties after medical school in France

Ranking	Specialty	Mean median rank	2017					2018				
			Median rank [IQR]	First	Last	Opened	Unfilled	Median rank [IQR]	First	Last	Opened	Unfilled
1	Infectious and tropical diseases	526.5	402 [179–1146]	1	3709	49	0	651 [154–1690]	1	3209	51	0
2	Ophtalmology	603.75	578 [294–1028]	3	2158	129	0	629 [275–1027]	18	1830	150	0
3	Plastic, reconstructive and aesthetic surgery	723	675 [411–929]	29	1553	27	0	771 [440–1390]	80	2177	29	0
4	Nephrology	734.25	748 [297–1622]	15	4395	76	0	720 [258–2087]	10	3791	80	0
5	Cardiovascular medicine	839.75	795 [364–1532]	4	2887	170	0	884 [407–1637]	23	2678	181	0
6	Dermatology-Venereology	1096	1091 [487–1556]	33	2247	90	0	1101 [482–1459]	11	2448	95	0
7	Radiology and medical imaging	1229	1192 [690–1818]	8	2536	245	0	1266 [667–1923]	3	3076	254	0
8	Hematology	1444	1461 [483–4228]	22	5628	44	0	1427 [539–3546]	86	5790	44	0
9	Oncology	1499.5	1479 [870–2461]	39	4146	117	0	1520 [736–2526]	12	3935	120	0
10	Otorhinolaryngology - Craniofacial surgery	1501	1339 [929–2055]	174	3147	76	0	1663 [999–2344]	56	3254	81	0
11	Neurology	1645	1374 [636–2322]	16	3643	121	0	1916 [723–2981]	5	4280	127	0
12	Neurosurgery	1670.5	1204 [538–2246]	190	3986	21	0	2137 [1039–3724]	228	5595	25	0
13	Maxillofacial surgery	1780.75	2002 [946–2445]	140	2870	24	0	1559 [1133–2043]	311	3250	27	0
14	Intensive care medicine	1811.75	1366 [615–2648]	107	4271	64	0	2257 [832–4177]	49	5318	72	0
15	Hepato-Gastroenterology	1883.75	1720 [771–2697]	71	3971	122	0	2047 [937–2779]	6	4332	128	0
16	Internal medicine and clinical immunology	1898.5	1831 [477–4135]	2	5693	113	0	1966 [652–4059]	7	6801	123	0
17	Orthopedic and trauma surgery	1913.5	1977 [1366–2783]	170	3467	116	0	1850 [1157–2712]	2	3887	121	0
18	Urology	1997.25	2075 [1124–3129]	224	3888	61	0	1919 [906–2966]	44	4088	62	0
19	Rheumatology	2081.5	1916 [1298–2730]	63	4290	83	0	2247 [1524–3171]	94	4159	86	0
20	Anaesthesiology and intensive care	2103.5	2165 [1162–2972]	24	4083	445	0	2042 [1081–2963]	25	4079	465	0
21	Nuclear medicine	2311	2080 [1635–2746]	314	3122	31	0	2542 [1808–3181]	268	3797	33	0
22	Pulmonology	2614	2346 [1400–3531]	129	4624	116	0	2882 [2011–3788]	8	4557	121	0
23	Obstetrics and gynecology	2757	2614 [1849–3474]	204	4538	197	0	2900 [2039–3747]	61	4843	204	0
24	Oral surgery	2808.5	2613 [1986–3013]	1520	3191	12	0	3003 [2115–3258]	1989	4127	12	0
25	Pediatrics	3150.25	2950 [1776–3915]	83	5129	316	0	3350 [1897–4401]	96	6141	330	0
26	Gynecology	3157.25	2985 [2271–3658]	564	4600	64	0	3329 [2390–4279]	347	5331	81	0
27	Pediatric surgery	3184.75	3157 [2574–3606]	701	4103	24	0	3212 [1926–3863]	918	5169	22	0
28	Pathology	3330	2611 [1212–3350]	31	4199	56	0	4049 [2461–4970]	582	5611	60	0
29	Gastro-enterologic surgery	3398.5	3265 [2423–3755]	121	4509	77	0	3532 [2462–4368]	441	5247	80	0
30	Vascular medicine	3678.75	3657 [3116–4115]	735	4635	44	0	3700 [3122–4134]	559	4633	46	0
31	Thoracic and cardiovascular surgery	3761	3804 [2761–4148]	807	4510	25	0	3718 [2482–5094]	217	5624	24	0
32	Vascular surgery	3765	3615 [2740–3942]	640	4439	29	0	3915 [3172–4167]	1980	4505	28	0
33	Endocrinology. diabetes and nutrition	4120.5	4061 [2068–4685]	166	5752	80	0	4180 [3128–5380]	375	6667	82	0
34	Physical medicine and rehabilitation	4236.75	4128 [2695–4968]	569	5926	94	0	4345 [3267–5068]	73	7068	99	0
35	Allergology	4736.5	4478 [3614–5277]	2055	6399	27	0	4995 [3949–5602]	2367	6682	28	0
36	Medical genetics	5354.75	3717 [2120–4737]	582	5801	20	0	6992 [3750–7690]	57	8470	20	0
37	Emergency medicine	5662.75	5454 [4374–6677]	331	8285	460	0	5871 [4631–7120]	1103	8693	469	21
38	Legal medicine and medical jurisprudence	5795.75	5768 [4936–6665]	1187	7441	26	0	5823 [4976–6368]	1500	7618	27	0
39	General medicine	5846	5812 [4346–6969]	50	8372	3132	187	5880 [4401–7140]	62	8706	3268	163
40	Psychiatry	6024.5	5773 [4250–7217]	84	8344	494	8	6276 [4504–7584]	495	8685	528	18
41	Geriatrics	6128	6164 [4714–7550]	275	8371	200	29	6092 [3984–7345]	72	8684	199	36
42	Public Health	6681.5	6256 [4055–7589]	390	8211	84	5	7107 [6325–8179]	1063	8674	90	21
43	Clinical biology	6860.25	6212 [5081–7556]	244	8363	110	0	7508 [6356–8065]	1366	8695	111	20
44	Occupational medicine	7698.5	7572 [6264–8037]	1012	8370	137	48	7825 [6903–8282]	3012	8699	129	48

### Survey participants

The survey was sent by email to all the ID residents in France (*n* = 100). The response rate to the survey was 90%: 46 women and 44 men, median age of 25 years (IQR, 24–25.25) completed the survey (Table 2). Residents from every residency program of ID in France participated in the survey (supplementary material). The median delay between the beginning of the ID residency and the survey was 9 months (IQR 3–15). Among the residents who participated in the study, two (2%) dropped out of the ID residency program, one to join the general medicine residency because of the workload in ID and one who finally chose the orthopedic surgery residency after having hesitated between the two specialties for years.

**Table 2:** Demographic characteristics and professional expectations of participants

	Number (%) (*n* = 90)
Women	46 (51)
Median age (years) [IQR]	25 [24–25.25]
Year of national ranking exam	
2017	48 (53)
2018	42 (47)
Internship in ID department during medical school	
Yes	71 (79)
No	19 (21)
Professional experience abroad before the choice	
Yes	27 (30)
No	63 (70)
Final decision to choose ID specialty	
During medical school	39 (43)
After the national ranking exam	51 (57)
Hesitation with other specialties^a^	
Internal medicine and clinical immunology	31 (34)
Family medicine	18 (20)
Intensive care	18 (20)
Nephrology	17 (19)
Hematology	16 (18)
Hepato-gastro-enterology	8 (9)
Cardiology	7 (8)
Dermatology	6 (7)
Neurology	6 (7)
Oncology	6 (7)
Interest in the following ID topics	
Tropical diseases	61 (68)
Antimicrobial stewardship	58 (64)
Infections in immunocompromised patients	57 (63)
HIV and sexually-transmitted infections	52 (58)
Emerging infectious diseases	45 (50)
Community-acquired infections	42 (47)
Humanitarian medicine	42 (47)
Travel medicine	39 (43)
Health-acquired infections	26 (29)
Public health	17 (19)
Vaccinations	14 (16)
Viral hepatitis	8 (9)
Most interesting aspects of physicians’ work	
Diversity of the tasks	55 (76)
Possibility to cure patients	48 (67)
Challenging diagnosis	46 (64)
Teamwork	42 (58)
Patient-physician relationship	41 (57)
Teaching and mentoring	39 (54)
Scientific and research work	31 (43)
Helping vulnerable patients	30 (42)
Follow-up of outpatients	15 (21)
Prospective future activity	
Clinical activity	87 (97)
Teaching	50 (56)
Research	39 (43)
Prospective working structure	
University hospital	68 (76)
General hospital	63 (71)
Non-governmental organization	34 (38)
Research institute	19 (21)
Multidisciplinary health clinic	19 (21)
Private hospital	18 (20)
Governmental organization	18 (20)
Public health institute	8 (9)
Private surgery	6 (7)

^a^Only the specialties cited by more than 5 participants are reported

### Expectations and career plan

Seventy-one participants (79%) did an internship in an ID department during medical school and 27 participants (30%) had experience working abroad in a hospital or a non-governmental organization (NGO) during medical school (Table 2). Thirty-nine participants (43%) decided to choose the ID specialty during medical school and 51 participants (57%) took their decision after the national ranking exam. The participants mainly hesitated between ID and internal medicine and clinical immunology (*n* = 31, 34%), family medicine (*n* = 18, 20%), intensive care (*n* = 18, 20%), nephrology (*n* = 17, 19%) and hematology (*n* = 16, 18%).

When asked about where they wanted to work in the future, the participants cited academic hospitals (*n* = 68, 76%), general hospitals (*n* = 63, 71%), NGOs (*n* = 34, 38%), research institutes (*n* = 19, 21%), multidisciplinary health clinic (*n* = 19, 21%), private hospitals (*n* = 18, 20%), governmental organizations (*n* = 18, 20%), public health institute (*n* = 8, 9%) and private surgeries (*n* = 6, 7%). Concerning their future position, 87 participants (97%) declared that they wanted to have a clinical activity, 50 participants (56%) a teaching activity and 39 participants (43%) a research activity.

Tropical medicine (*n* = 61, 68%), antimicrobial stewardship (*n* = 58, 64%), infections of immunocompromised patients (*n* = 57, 63%) and HIV and sexually transmitted infections (*n* = 52, 58%) were the areas of ID that interested the most participants. Viral hepatitis (*n* = 8, 9%), vaccinations (*n* = 14, 16%) and public health (*n* = 17, 19%) were the areas of ID that drew the least interest among ID residents.

### Motivating factors

**Fig. 2:**
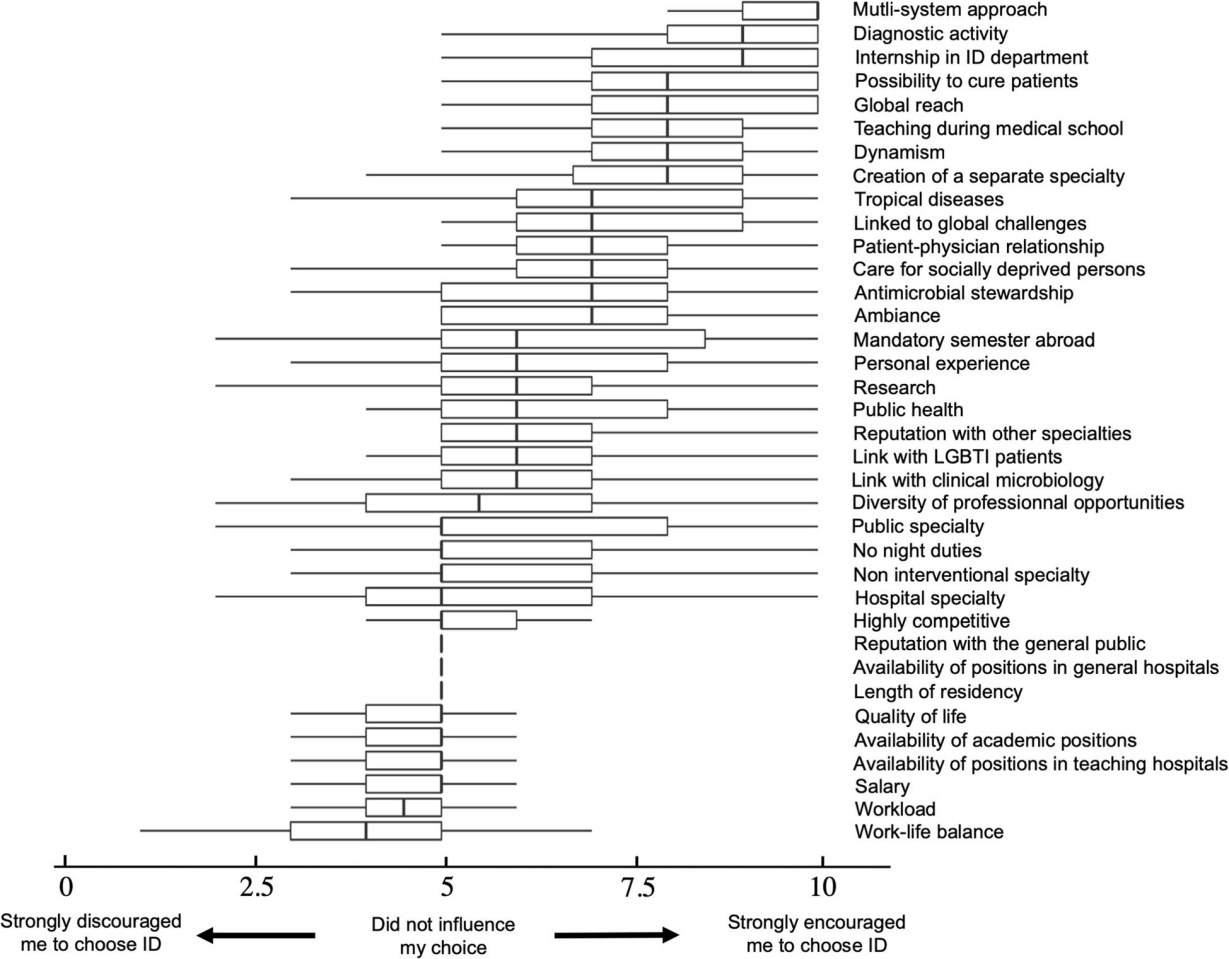
Motivations and reservations to choose ID specialty. Legend: Boxplots of the 36 factors that were evaluated by ID residents. The residents had to use a slider to say if this factor encouraged them to or discouraged them from choosing ID. The slider position was attributed a figure between 0 (strongly discouraged) to 10 (strongly encouraged). The lower and upper hinges correspond to the first and third quartiles. The upper and lower whisker extends from the hinge to the largest value no further than 1.5 x IQR from the hinge. The bold lines correspond to the medians

The main motivations to choose ID were (median [IQR]): the multi-system approach of the specialty (10 [9, 10]), the importance of diagnostic medicine in the specialty (9 [8–10]), having done an internship in ID during medical school (9 [7–10]), the fact that ID is a specialty where patients can generally be cured (8 [7–10]) and the global reach of ID (8 [7–10]) (Fig. 2). The creation of a qualifying specialty was a motivating factor (8 [6.75–9]) as well as the quality of the teaching of ID during medical school (8 [7–9]), the dynamism (8 [7–9]) and ambiance (7 [5–8]) in the specialty and the mandatory semester abroad that has been included in the French ID residency (6 [5–8.5]). The participants said that the work-life balance (4 [3–5]), the workload (4.5 [4, 5]), the salary (5 [4, 5]), the availability of clinical positions in teaching hospitals (5 [4, 5]), the availability of academic positions (5 [4, 5]) and the quality of life (5 [4, 5]) were potential deterrents. The participants were given the opportunity to add any other factors that influenced their choice: 2 participants cited the “prestige” of the specialty as a motivating factor.

### Measures to increase the attractiveness of ID

**Fig. 3:**
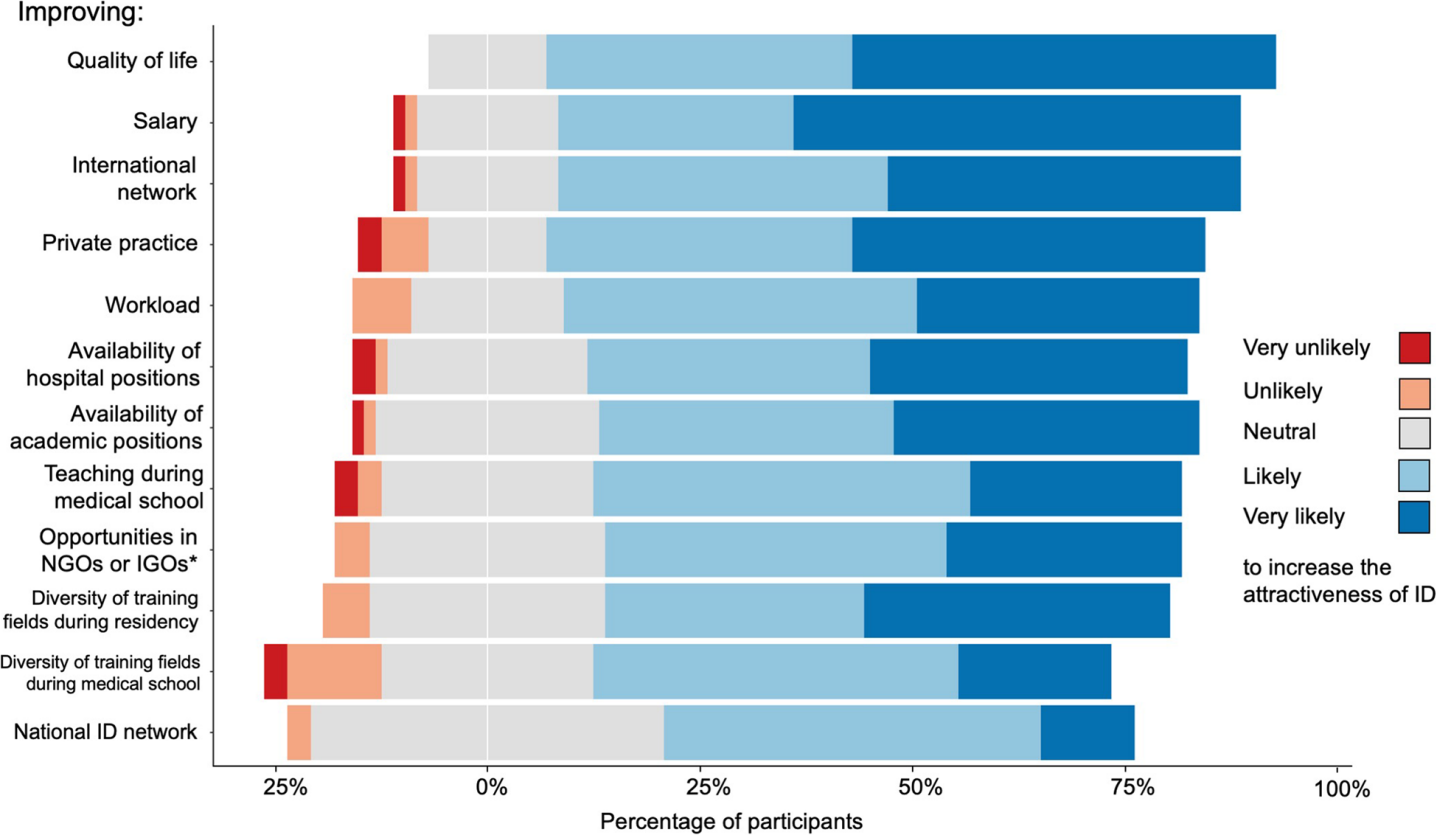
Could the following measures increase the attractiveness of infectious diseases specialty?. Legend: Likert scale of the 12 potential measures to increase the attractiveness of the specialty evaluated by ID residents. Each bar represents 100% of the participants and is ranked from most likely to least likely. *: Non-governmental organizations or Intergovernmental organizations (e.g. the United Nations)

Improving the quality of life of ID physicians was judged very likely or likely to increase the attractiveness of the specialty by 86% of participants, increasing the salary by 81%, developing the international network by 81%, developing private practice in ID by 78%, decreasing the workload by 75%, increasing the availability of non-academic hospital positions by 71% and increasing the availability of academic positions by 71% (Fig. 3). Overall, the 12 measures evaluated were judged very likely or likely to increase the attractiveness of the ID specialty by more than half the participants.

## Discussion

This study brings interesting results for the choice of the ID specialty at a national level. The two most motivating factors to choose ID were the multi-system approach of the specialty and its diagnostic activity. These results indicate that ID should keep its strong internal medicine roots with a diversity of activities instead of moving toward overspecialization. The quality of the teaching of ID as well as an internship in ID during medical school boosted the choice of the specialty. These results are similar to other studies in which ID education and early ID rotation were associated with a career interest in ID [15, 16]. The international and global reach of ID specialty have been consistently cited as motivating and more than 80% of the participants thought that the international network should be strengthened. International collaborations are important assets of the specialty and we should increase our effort to build bridges between national and continental societies of ID. The decision to include a mandatory semester abroad in the French ID residency is a step toward this aim and was deemed motivating by most of the participants.

The fact that ID is currently the most popular specialty in France differs from what happens in other countries such as the USA [7]. Yet the deterrents that we identified in our study are really close to the ones identified in previous studies: concern about salary, the work-life balance and limited job availability [12]. While trainees in the USA are probably not more driven by money than in France, the impact of salary on the choice of specialty is probably lower in France than in the USA. Indeed, > 86% of medical students in the US graduate with debt (approximately $120,000/student) [17], whereas French medical education is supported by the government and costs around $500 per year. As ID physicians are among the lowest paid physicians in the USA [18], student debts may have a higher potential to influence medical students than in France. An increase of the salary of ID physicians in France was still described in our survey as one of the likeliest measures to increase the attractiveness of ID. Recommendations for working toward appropriate compensation for ID physicians have been published and include suggestions such as awarding financial compensation for ID who work in the public service or ensuring that the financial compensation for ID physicians reflects the added value to public health [2]. Studies have reported high rates of burnout symptoms among ID physicians [19, 20] and our results confirm that the concerns about the quality of life, the workload and the work-life balance are potential deterrents from choosing ID. The need to improve the work-life balance of physicians is part of the global healthcare system and have to be tackled at the national and political level. To achieve this aim, the ID community probably needs to develop a common advocacy for the ID specialty directed at policy makers and the general public. This research was done before the COVID-19 outbreak but this pandemic further highlights the need to support and encourage students and residents who want to become ID specialists as they are and will be increasingly needed to organize the response to existing and future infectious threats [21].

According to our results, most ID residents want to have a clinical activity (97%) and a teaching activity (56%) but less than half want to participate in research (43%). Scientific research is particularly important in ID with global challenges such as emerging diseases and antimicrobial resistance. The fact that ID physicians in France have an important clinical workload and do not have a dedicated time for research may be an important barrier to the motivation to participate in research [22]. Increasing early research opportunities in ID has been cited as a potential measure toward a reinvigorated interest in the specialty [23, 24]. Tropical medicine and antimicrobial stewardship were the topics that interested the most the participants showing the need for a strong and sustained support for international collaboration and antimicrobial stewardships programs [23], but only 16% of the participants in our study expressed an interest in vaccination. This finding is worrying, especially with the rise of vaccine hesitancy globally and particularly in France [25, 26], however this might be due to the fact that vaccination is a clinical area shared with family medicine and public health.

In this study, we did not contact residents in other specialties and thus cannot analyze the reasons why other students did not choose ID. Moreover, this is a study done in France in a specific healthcare context and only 2 years after the recognition of ID as a qualifying specialty. The network of young ID doctors that is behind this work has been created approximately at the same time as the ID specialty and thus we cannot compare our results with previous data. Our results may not reflect the situation in other countries, especially countries in which medical studies are expensive or countries in which the healthcare system is mainly private. However, this study validates most of the potential measures that have been suggested in previous studies in order to increase the attractiveness of ID, suggesting that our results are not limited to France [12, 13, 23].

## Conclusion

The recognition of ID as a qualifying specialty in France can be considered a success insofar as the specialty reached first rank among all medical and surgical specialties. Individuals who choose ID residency are attracted by the prospect of intellectual stimulation inherent in the specialty (multi-system approach, diagnostic challenges), its global reach and its variety of activities but have reservations regarding the salary, the workload and the availability of positions mainly in university hospitals. According to this national survey, the decision to make ID a qualifying specialty in France strongly encouraged the students to choose ID. These results enable us to be optimistic about the future of this essential specialty and may encourage some countries to recognize ID as a qualifying specialty.

## Supplementary Information

**Additional file 1.** Supplementary material: map of participating programs.

**Additional file 2.** Supplementary material: questionnaire.

## Data Availability

The datasets used and/or analysed during the current study are available from the corresponding author on reasonable request.

## Abbreviations

CNIL: French data protection authority
HIV: Human Immunodeficiency Virus
ID: Infectious and tropical Diseases
IQR: Interquartile Range
NGO: Non-Governmental Organization
RéJIF: Young French Infectious Diseases Physicians’ Network
SPILF: French Society for Infectious Diseases
USA: United States of America
