# Supplementary material for: How and why do French medical students choose the specialty of infectious and tropical diseases? A national cross-sectional study
Source: BMC Med Educ. 2020 Oct 31;20:397. doi: 10.1186/s12909-020-02317-9 (PMC7602756; doi:10.1186/s12909-020-02317-9)
Supplement: Supplementary file 1 — Additional file 1. Supplementary material: map of participating programs. [file 12909_2020_2317_MOESM1_ESM.docx]

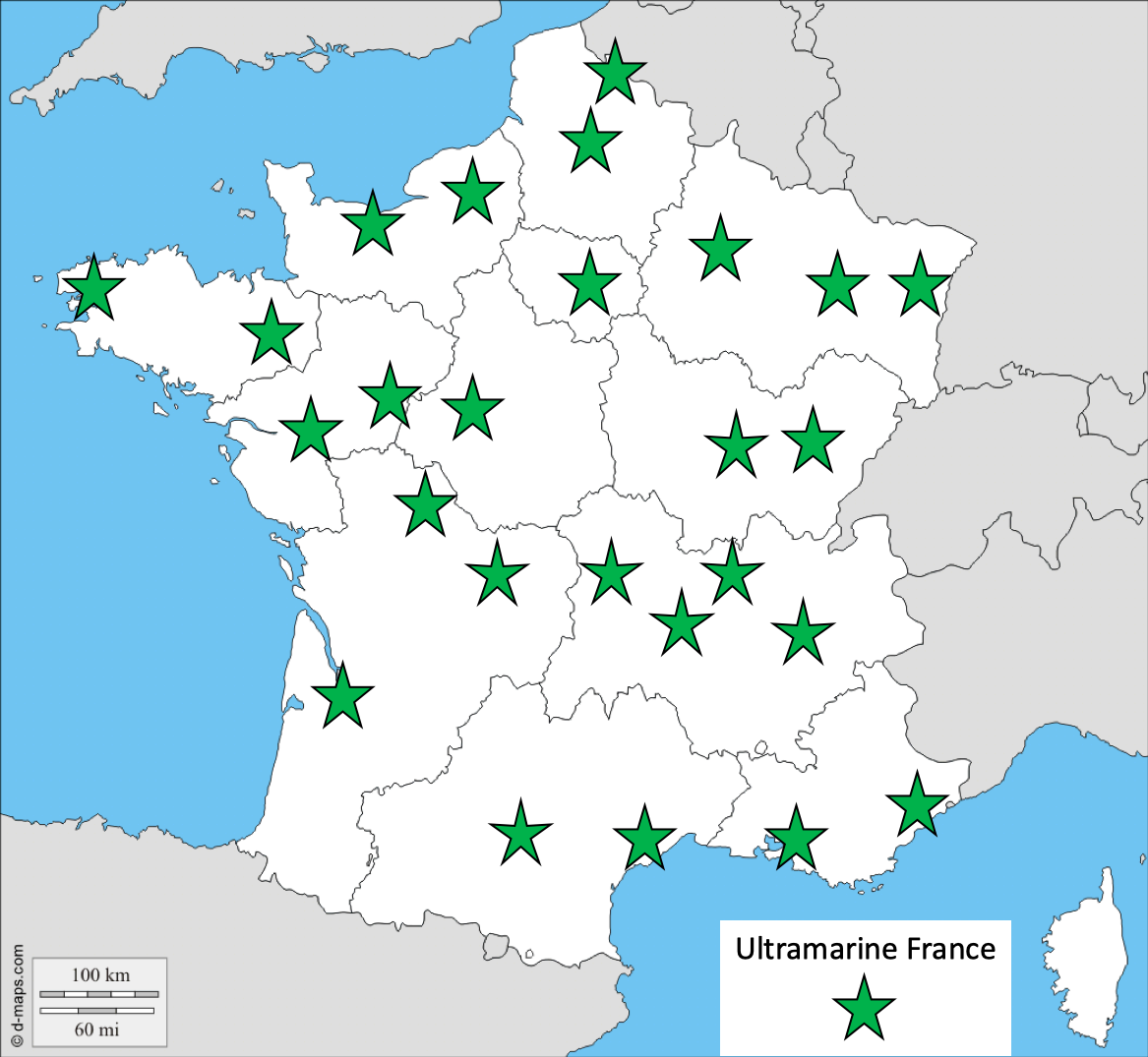


Supplemental Material: Map Legend

All 28 ID residency programs in France were asked for survey participation.
Star = participated

Circle = did not participate
