# Supplementary material for: How and why do French medical students choose the specialty of infectious and tropical diseases? A national cross-sectional study
Source: BMC Med Educ. 2020 Oct 31;20:397. doi: 10.1186/s12909-020-02317-9 (PMC7602756; doi:10.1186/s12909-020-02317-9)
Supplement: Supplementary file 2 — Additional file 2. Supplementary material: questionnaire. [file 12909_2020_2317_MOESM2_ESM.doc]

General data

**The specialty of Infectious and Tropical Diseases (ID) is one of the top specialties in France since the creation of the ID residency, which is not the case in all countries. The French Young ID Physicians Network proposes this survey to describe and understand your motivations to choose the residency of Infectious and Tropical Diseases.**

**The first page of this survey collects general and demographic information and the second page concerns more particularly your motivations for choosing this specialty.**

**ALL ANSWERS WILL BE ANONYMOUS. ACCESS TO DATA IS PROTECTED AND DATA WILL BE DESTROYED AFTER ANALYSIS. Questions with asterisks require an answer.**

1. When did you attend the national ranking exam?

2017

2018

Other (Please precise)

2. What is your gender?

Female

Male

Other

1. How old are you?
2. Which city did you choose?
3. When did you first develop an interest for ID?

After the national ranking exam 2nd/3rd year of medical school

6th year of medical school, before the national ranking exam 1st year of medical school

5th year of medical school In secondary school

4th year of medical school Before secondary school

1. When did you take the final decision to choose ID?

After the national ranking exam 2nd/3rd year of medical school

6th year of medical school, before the national ranking exam 1st year of medical school

5th year of medical school In secondary school

4th year of medical school Before secondary school

7. Did you do an internship in ID during medical school?

Yes

No

8. Have you had a professional experience abroad?

Yes

No

Please precise:

1. Choose the topics in ID that interest you?

|  |  |  |  |  |
| --- | --- | --- | --- | --- |
| Antimicrobial stewardship | |  | Community-acquired infection | |
|  | |  |  |  |
|  | | |  |  |
|  |  |  | Humanitarian medicine | |
| Public health | |  | Travel medicine | |
|  |
|  |  |  |
| HIV and sexually transmitted infections | |  | Vaccination | |
|  |
|  |  |  |
| Viral hepatitis | |  | Health-acquired infections | |
|  |
|  |  |  |
| Tropical diseases | |  | Emerging diseases | |
|  |
|  |  |  |
| Infectious diseases of immunocompromised patients | |  |  |  |
|  |  |  |
| Other (please precise) | | |  |  |

1. Among the following persons please tick those who have given you an interest in the specialty of infectious and tropical diseases:

in ID In another specialty

A fellow

A professor or associate professor

An attending physician

A resident

A patient

Other (please precise

11. Which activities would you like to have in your future position?

Clinical activity

Research activity

Teaching activity

12. Where would you like to work following residency?

General hospital

Teaching hospital

Pluridisciplinary health clinic

Private hospital

Private surgery

Non-governmental organization

Governmental organization (Minister of Health, United nations, etc.)

Research institute

Public health institute

Other (Please precise)

13. According to you, what is the reputation of the ID specialty among other specialties?

Very bad Average Very good


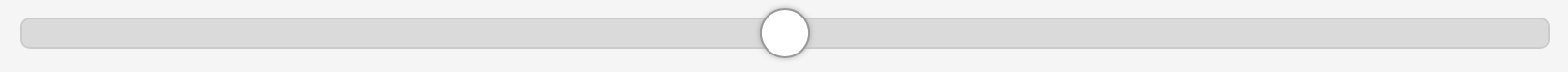

14. According to you, what is the reputation of the ID specialty among the general public?

Very bad Average Very good


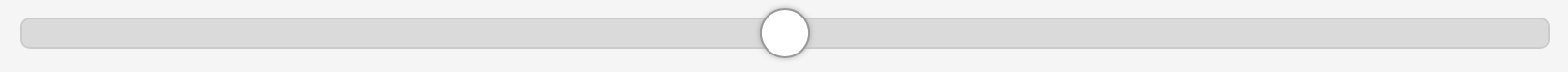


15. According to you, how is the quality of life in ID?

Very bad Average Very good


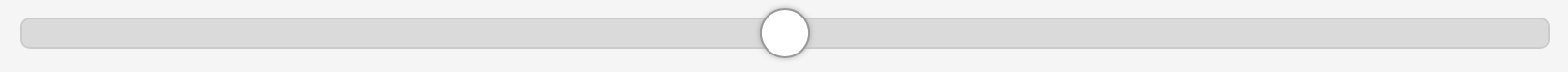


16. According to you, how is the workload in ID?

Very low Average Very high


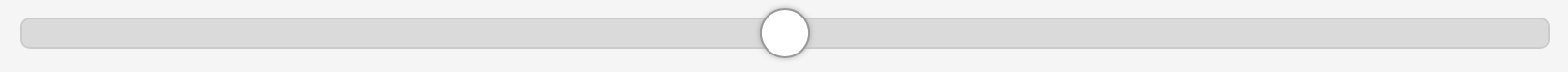


1. According to you, how is the work-life balance in ID?

Very bad balance Average Very good balance


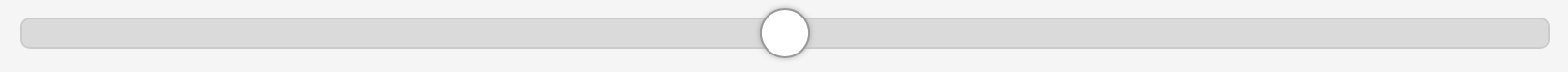


18. What do you think of the ambiance in ID?

Very bad ambiance Average Very good ambiance


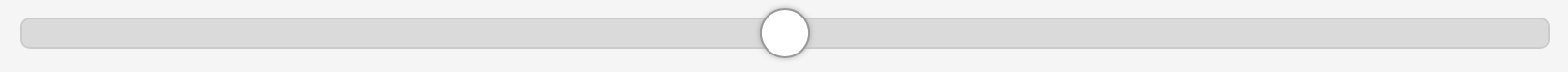


19. What do you think of the dynamism of the ID specialty?

Not dynamic at all Average Very dynamic


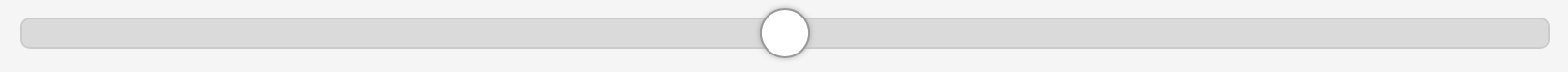


1. What do you think of the professional opportunities following your ID residency?

Narrow Average Very broad


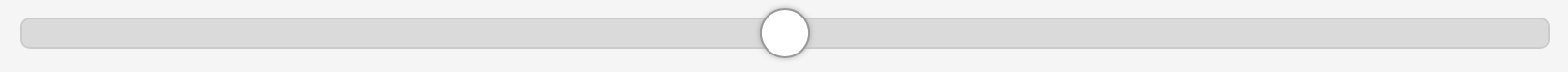


1. According to you, what is the availability of academic positions in ID?

Very few available positions Average number of available positions Many available positions


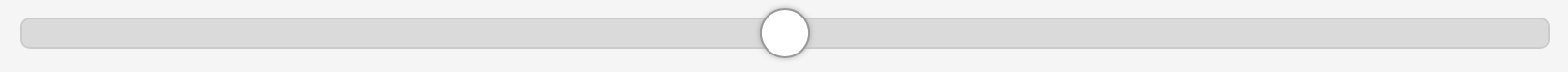


1. According to you, what is the availability of non-academic positions in teaching hospitals in ID?

Very few available positions Average number of available positions Many available positions


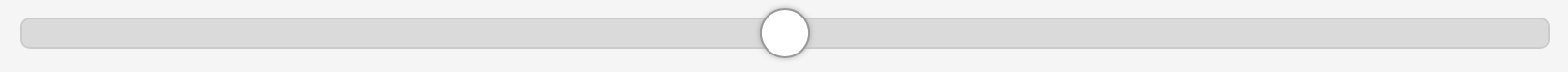


1. According to you, what is the availability of positions in general hospitals in ID?

Very few available positions Average number of available positions Many available positions


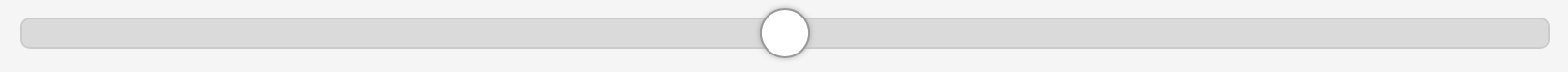


24. What do you think of the salary of ID physicians?

Very low Average Very high


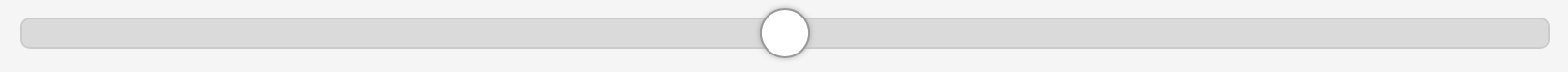


1. What is your estimate of the mean salary of an ID attending physicians at mid-career?

26.Did you hesitate with other specialties?

Specialty 1

Specialty 2

Specialty 3

Specialty 4

Specialty 5

Motivations and reservations

**For each of the following factors, you need to position the cursor according to the role it played in your choice of Infectious Diseases and Tropical Diseases. If the cursor is to the left, it means that this factor has strongly discouraged to choose ID, if it is on the far right it means that this factor has strongly encouraged you to choose ID.**

**ALL ANSWERS WILL BE ANONYMOUS. ACCESS TO DATA IS PROTECTED AND DATA WILL BE DESTROYED AFTER ANALYSIS. Questions with asterisks require an answer.**

1. The teaching of ID during medical school:

Strongly discouraged Did not play a role Strongly encouraged
me to choose ID in my choice me to choose ID


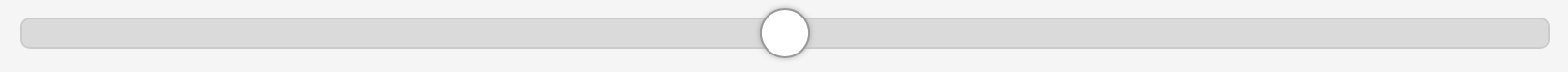


2. Doing an internship in ID during medical school:

Strongly discouraged Did not play a role Strongly encouraged
me to choose ID in my choice me to choose ID


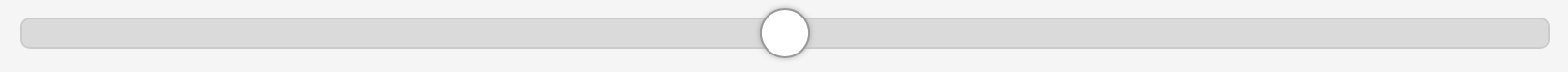


3. Scientific research in ID:

Strongly discouraged Did not play a role Strongly encouraged
me to choose ID in my choice me to choose ID


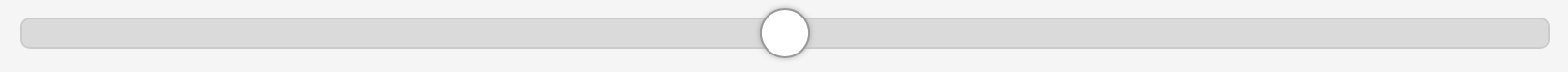


4. The link of ID with global challenges:

Strongly discouraged Did not play a role Strongly encouraged
me to choose ID in my choice me to choose ID


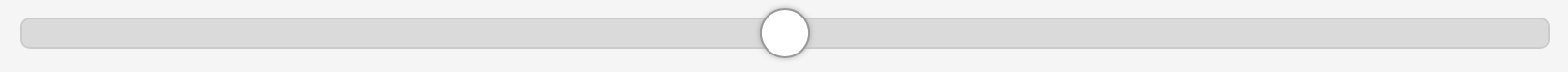


5. The mean salary of ID physicians:

Strongly discouraged Did not play a role Strongly encouraged
me to choose ID in my choice me to choose ID


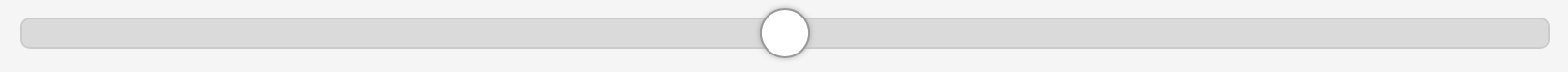


6. The links between ID and public health:

Strongly discouraged Did not play a role Strongly encouraged
me to choose ID in my choice me to choose ID


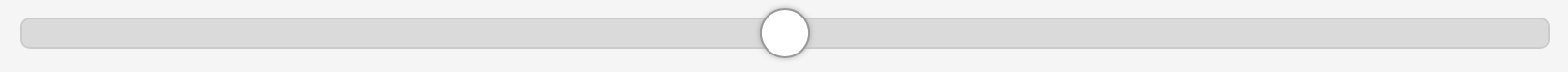


7. The links between ID and the care for socially deprived persons:

Strongly discouraged Did not play a role Strongly encouraged
me to choose ID in my choice me to choose ID


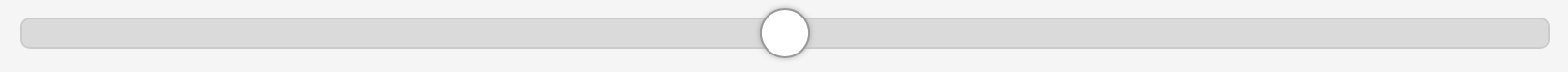


8. Tropical diseases:

Strongly discouraged Did not play a role Strongly encouraged
me to choose ID in my choice me to choose ID


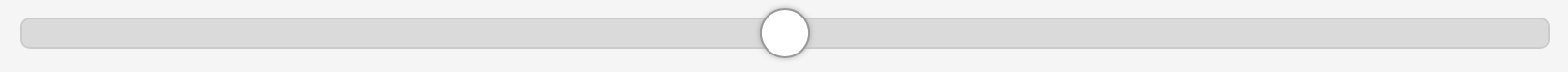


9. The reputation of ID among other specialties:

Strongly discouraged Did not play a role Strongly encouraged
me to choose ID in my choice me to choose ID


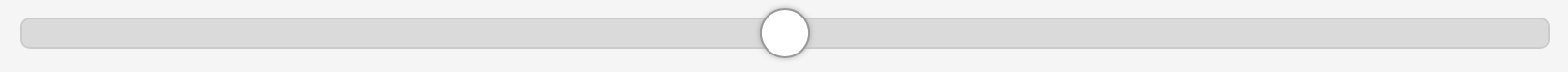


10. The reputation of ID among the general public

Strongly discouraged Did not play a role Strongly encouraged
me to choose ID in my choice me to choose ID


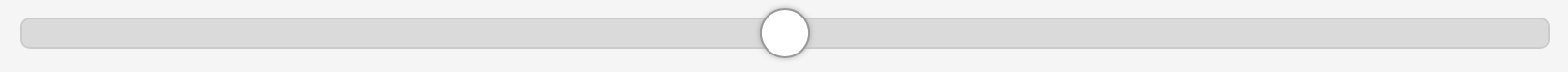


11. The work-life balance in ID:

Strongly discouraged Did not play a role Strongly encouraged
me to choose ID in my choice me to choose ID


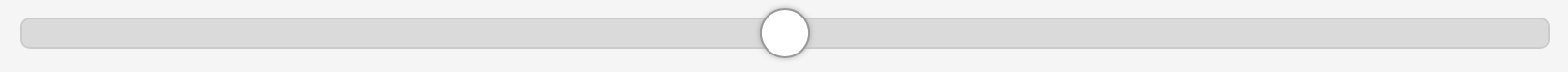


12. The quality of life in ID

Strongly discouraged Did not play a role Strongly encouraged
me to choose ID in my choice me to choose ID


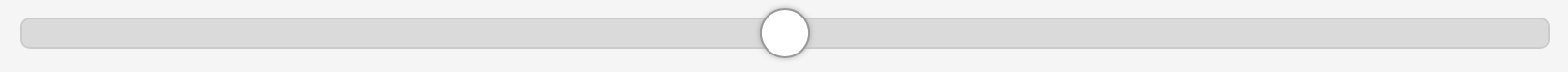


13. The ambiance in ID:

Strongly discouraged Did not play a role Strongly encouraged
me to choose ID in my choice me to choose ID


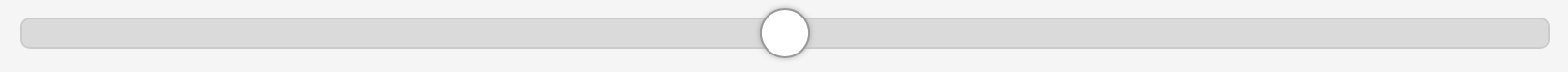


14. The fact that ID is mainly a non interventional specialty:

Strongly discouraged Did not play a role Strongly encouraged
me to choose ID in my choice me to choose ID


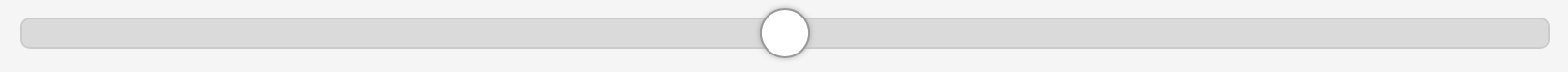


15. The global reach of ID and the internation network:

Strongly discouraged Did not play a role Strongly encouraged
me to choose ID in my choice me to choose ID


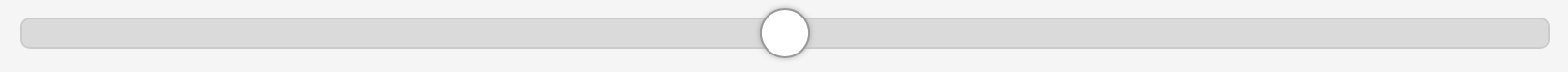


16. The fact that there is a mandatory semester abroad during residency:

Strongly discouraged Did not play a role Strongly encouraged
me to choose ID in my choice me to choose ID


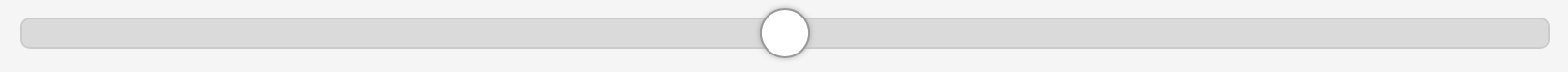


17. The links between the ID specialty and the LGBTI+ community

Strongly discouraged Did not play a role Strongly encouraged
me to choose ID in my choice me to choose ID


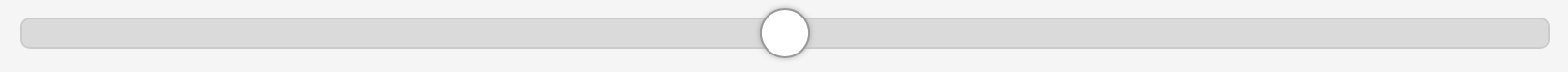


18. The systemic aspect of ID:

Strongly discouraged Did not play a role Strongly encouraged
me to choose ID in my choice me to choose ID


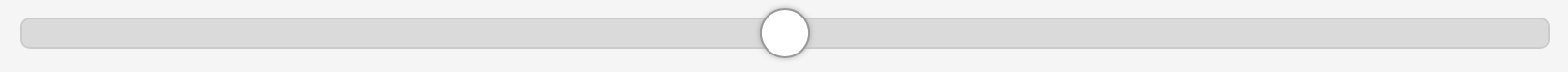


19. The workload in ID:

Strongly discouraged Did not play a role Strongly encouraged
me to choose ID in my choice me to choose ID


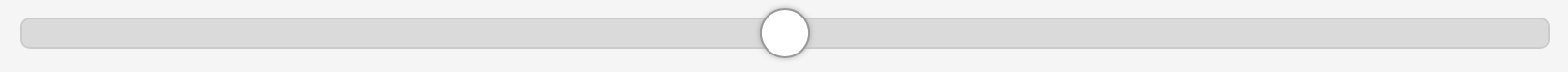


20. The fact that ID is mainly a public specialty:

Strongly discouraged Did not play a role Strongly encouraged
me to choose ID in my choice me to choose ID


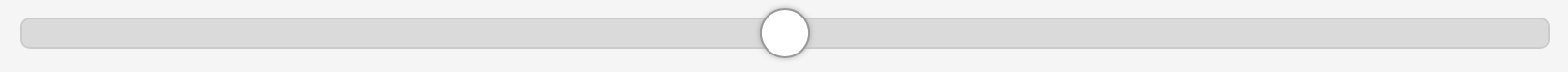


21. The fact that ID is mainly a hospital specialty:

Strongly discouraged Did not play a role Strongly encouraged
me to choose ID in my choice me to choose ID


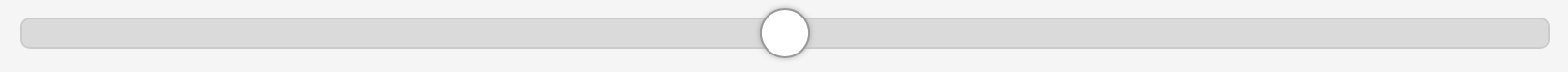


22. The availability of academic positions in ID

Strongly discouraged Did not play a role Strongly encouraged
me to choose ID in my choice me to choose ID


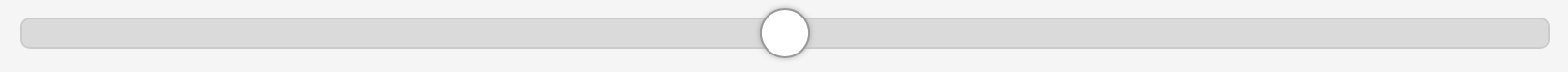


23. The availability of non-academic positions in teaching hospitals in ID:

Strongly discouraged Did not play a role Strongly encouraged
me to choose ID in my choice me to choose ID


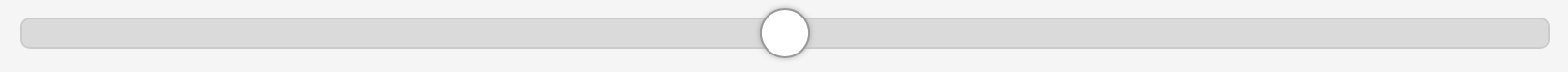


24. The availability of ID positions in general hospitals

Strongly discouraged Did not play a role Strongly encouraged
me to choose ID in my choice me to choose ID


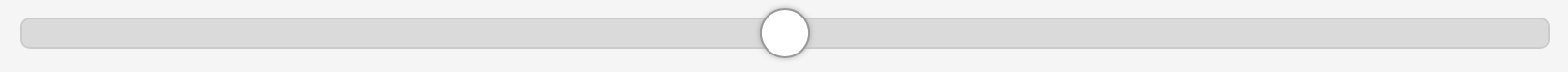


25. The fact that ID is a specialty with mainly patients who can be cured:

Strongly discouraged Did not play a role Strongly encouraged
me to choose ID in my choice me to choose ID


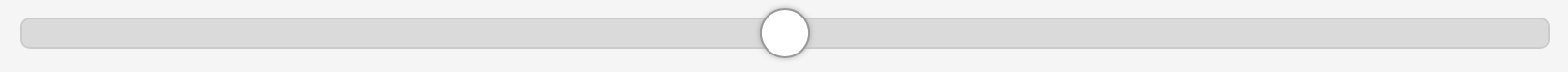


26. The length of the residency (5-years)

Strongly discouraged Did not play a role Strongly encouraged
me to choose ID in my choice me to choose ID


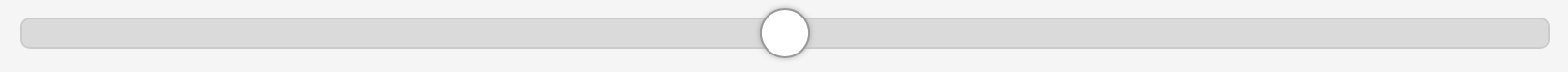


27. The possibility to work in an antimicrobial stewardship team:

Strongly discouraged Did not play a role Strongly encouraged
me to choose ID in my choice me to choose ID


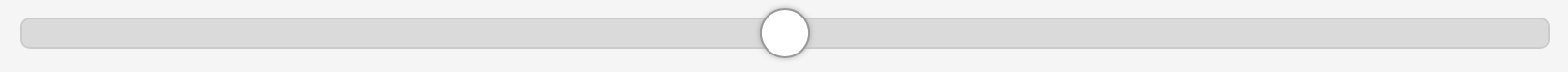


28. The dynamism in ID:

Strongly discouraged Did not play a role Strongly encouraged
me to choose ID in my choice me to choose ID


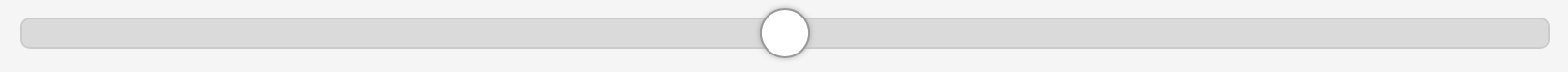


29. The fact that ID is a highly competitive specialty:

Strongly discouraged Did not play a role Strongly encouraged
me to choose ID in my choice me to choose ID


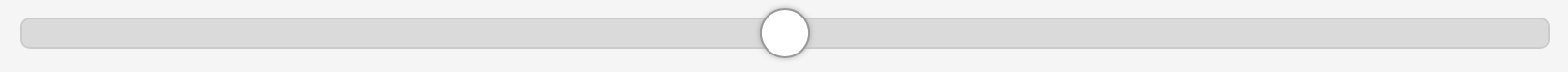


30. The diversity of professionnal opportunities following residency:

Strongly discouraged Did not play a role Strongly encouraged
me to choose ID in my choice me to choose ID


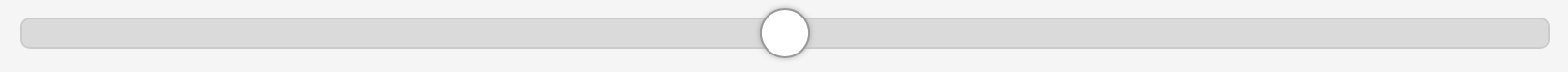


31. The link with clinical microbiology:

Strongly discouraged Did not play a role Strongly encouraged
me to choose ID in my choice me to choose ID


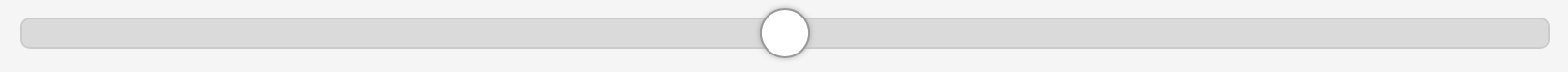


32. The fact that ID is a diagnostic specialty:

Strongly discouraged Did not play a role Strongly encouraged
me to choose ID in my choice me to choose ID


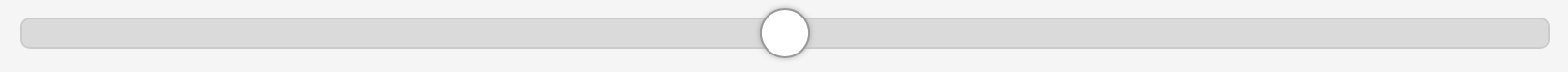


33. The importance of the patient-physician relationship in ID:

Strongly discouraged Did not play a role Strongly encouraged
me to choose ID in my choice me to choose ID


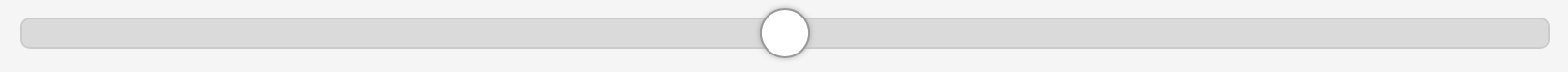


34. The creation of a residency in ID:

Strongly discouraged Did not play a role Strongly encouraged
me to choose ID in my choice me to choose ID


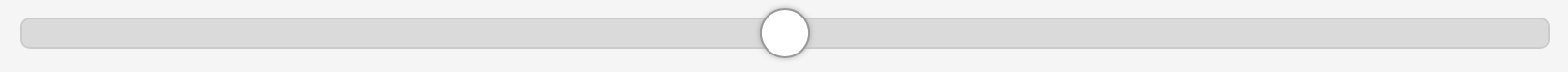


35. The absence of night duties in ID:

Strongly discouraged Did not play a role Strongly encouraged
me to choose ID in my choice me to choose ID


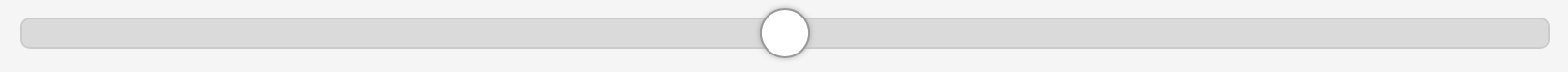


- 36. Would the following measures increase the attractiveness of id?

Very unlikely Unlikely Neutral Likely Very likely

Improving the teaching of ID during medical school

Diversification of the training fields in ID during medical school

Decreasing the workload in ID

Increasing the availability of academic positions in ID

Increasing the availability of non-academic positions in public hospital in ID

Increasing the salary of ID specialists

Increasing the possibility to work in the private sector in ID

Improving the quality of life in the specialty

Increasing the availability of positions in NGOs and governemental and intergovernmental organizations

Developing the international network in ID

Meilleure structuration du réseau des infectiologues

Diversification of the training fields during ID residency

37. What do you like the most in the ID practice?

| To diagnose a complex disease | |  | Teaching and mentoring | |
| --- | --- | --- | --- | --- |
| To cure patients | |  | To care for socially deprived persons | |
|  |
|  |
| To follow outpatients | |  | The scientific research | |
|  |
|  |
| The patient-physician relationship | |  | The diversity of the tasks | |
|  |
|  |
| The teamwork | |  |  |  |
|  |  |  |
| Other (Please precise) | |  |  |  |
|  |  |  |  |  |
|  |  |  |  |  |

38. Do you have any other ideas to explain why the ID specialty is one of the top specialty to be chosen after the national ranking exam?

39. Do you have any comments on this survey?
